# AMPK‐mediated formation of stress granules is required for dietary restriction‐induced longevity in *Caenorhabditis elegans*

**DOI:** 10.1111/acel.13157

**Published:** 2020-05-20

**Authors:** Chen‐Ting Kuo, Guan‐Ting You, Ying‐Jie Jian, Ting‐Shin Chen, Yu‐Chen Siao, Ao‐Lin Hsu, Tsui‐Ting Ching

**Affiliations:** ^1^ Institute of Biopharmaceutical Sciences Yang‐Ming University Taipei Taiwan; ^2^ Institute of Biochemistry and Molecular Biology National Yang‐Ming University Taipei Taiwan; ^3^ Research Center for Healthy Aging and Institute of New Drug Development China Medical University Taichung Taiwan; ^4^ Division of Geriatric and Palliative Medicine Department of Internal Medicine University of Michigan Ann Arbor MI USA

**Keywords:** AMPK, dietary restriction, heat shock, HSF‐1, longevity, stress granule

## Abstract

Stress granules (SGs) are nonmembranous organelles that are dynamically assembled and disassembled in response to various stressors. Under stressed conditions, polyadenylated mRNAs and translation factors are sequestrated in SGs to promote global repression of protein synthesis. It has been previously demonstrated that SG formation enhances cell survival and stress resistance. However, the physiological role of SGs in organismal aging and longevity regulation remains unclear. In this study, we used TIAR‐1::GFP and GTBP‐1::GFP as markers to monitor the formation of SGs in *Caenorhabditis elegans*. We found that, in addition to acute heat stress, SG formation could also be triggered by dietary changes, such as starvation and dietary restriction (DR). We found that HSF‐1 is required for the SG formation in response to acute heat shock and starvation but not DR, whereas the AMPK‐eEF2K signaling is required for starvation and DR‐induced SG formation but not heat shock. Moreover, our data suggest that this AMPK‐eEF2K pathway‐mediated SG formation is required for lifespan extension by DR, but dispensable for the longevity by reduced insulin/IGF‐1 signaling. Collectively, our findings unveil a novel role of SG formation in DR‐induced longevity.

## Graphical abstract

In addition to heat stress, starvation and dietary restriction (DR) can activate stress granule (SG) formation in *Caenorhabditis elegans*. HSF‐1 and AMPK are two key regulators for the SG formations. HSF‐1 is required for the SG formation in response to acute heat shock and starvation but not DR, whereas the AMPK‐eEF2K pathway is required for starvation and DR‐induced SG formation but not heat shock. Furthermore, AMPK‐mediated SG formation contributes to DR‐induced longevity.

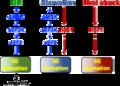

## 1 INTRODUCTION

Regulation of cellular homeostasis is pivotal for the survival of a cell. Highly conserved mechanisms have evolved which enable cells to cope with various environmental stresses that often disrupt cellular homeostasis. For instance, the global attenuation of protein translation that prevents cells from erroneous protein synthesis is a common phenomenon in response to stress conditions. Sequestration of nontranslating mRNAs into stress granules (SGs) is one such mechanism that attenuates protein synthesis during stress. Stress granules are cytosolic assemblies consisting of nontranslating mRNAs, small 40S ribosomes, mRNA‐associated translation initiation complexes, and RNA‐binding proteins (Anderson & Kedersha, 2006; Anderson, Kedersha, & Ivanov, 2015; Kedersha, Ivanov, & Anderson, 2013). It has been suggested that the SGs function as triage sites redirecting mRNAs to either translation, sequestration, or degradation (Anderson & Kedersha, 2008). Therefore, SG assembly represents a key role in protein and RNA homeostasis under adverse conditions and is a tightly regulated process. Not surprisingly, dysregulation of SG dynamic has recently been linked to various diseases, such as cancer, inflammatory, neurodegenerative, and neuromuscular diseases (Protter & Parker, 2016).

It has been reported that the phosphorylation of eukaryotic initiation factor 2α (eIF2α) is required for SG assembly in cells under stress conditions, such as heat shock and ultraviolet irradiation (Kedersha, Gupta, Li, Miller, & Anderson, 1999). eIF2α is a subunit of the heterotrimeric eIF2 complex that recruits initiator methionyl‐tRNA to the translation initiation complexes and mediates the start codon recognition. Upon stress, phosphorylation of eIF2α at the S51 residue prevents the recycling of eIF2 complex between successive rounds of protein synthesis, ultimately leading to a decrease in global translation (Clemens, 2001). Meanwhile, the accumulation of stalled translation preinitiation complexes would induce the assembly of stress granules. Although phosphorylation of eIF2α is a major trigger of SG formation, phospho‐eIF2α‐independent mechanisms of SG assembly have also been described. For example, both mTOR signaling and formation of the eIF4E complex are involved in promoting the formation of SG (Fournier et al., 2013).

During the initiation stage of SG formation, granule nucleating proteins assemble in the cytoplasm to produce initial foci. The proteins that facilitate the formation of this aggregation are RNA‐binding proteins (RBPs) that contain prion‐like (PLDs) or intrinsically disordered domains (IDDs). The primary RBPs in nucleating SG assembly are Ras GTPase‐activating protein‐binding protein 1 (G3BP1), T‐cell intracellular antigen‐1 (TIA‐1), TIA‐1‐related (TIAR), tristetraprolin (TTP), and fragile X mental retardation protein (FMRP) (Mahboubi & Stochaj, 2017). Recent studies have suggested that SGs are organized into a stable core that is embedded in a more dynamic shell. Both G3BP1 and TIAR have been confirmed as the mammalian SG core proteins by proteomic analysis (Jain et al., 2016). It has also been shown that the deletion of G3BP1 or TIAR impairs SG assembly in mammalian cells, indicating the substantive roles of these two RBPs in SG formation.

Complete removal of key SG core proteins often leads to embryonic lethality in animal studies. For example, homozygous null mutations of the *G3bp1, Tia1,* or *Tiar* gene in mice induce embryonic lethality (Sanchez‐Jimenez & Izquierdo, 2013; Zekri et al., 2005). Therefore, the physiological roles of SG other than those in embryonic development, such as stress response or longevity regulation, remain less explored at the organismal level. It has been previously reported that loss of *tiar‐1*, a worm homolog of mammalian TIA‐1/TIAR, impairs oogenesis in *Caenorhabditis elegans* (Silva‐Garcia & Estela Navarro, 2013). Recent studies suggested that *tiar‐1* may protect germ cells from heat shock stress (Huelgas‐Morales, Silva‐Garcia, Salinas, Greenstein, & Navarro, 2016). Similar to the observations in mammalian cells, the formation of SG in worms has been reported to be induced by different environmental stressors, such as heat or oxidative stress, using GFP::TIAR‐1 as a reporter (Lechler et al., 2017; Rousakis et al., 2014). Furthermore, stress‐induced SGs disperse in the cytoplasm within a short period of recovery. However, whether SG may form in response to dietary changes at the organismal level remains mostly unexplored. In this study, we first investigated SG formation under different dietary conditions. We then examined how different signaling pathways regulate SG formation and whether SG formation is involved in longevity regulation in *C. elegans*. Our study has revealed that dietary restriction and starvation could activate intestinal SG formation via AMPK‐eEFK2 signaling pathway, while acute heat shock and starvation might do so via HSF‐1 pathway in *C. elegans.* Furthermore, we have found that the AAK‐1‐mediated SG formation is required for DR‐induced longevity.

## 2 RESULTS

### 2.1 Acute heat stress, starvation, and dietary restriction could induce stress granule formation in * Caenorhabditis elegans*

**FIGURE 1:**
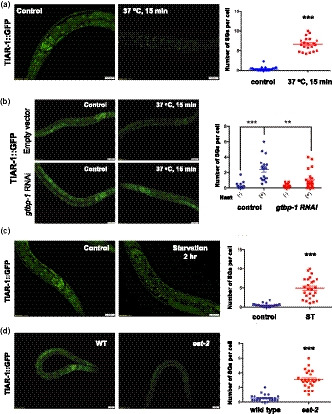
The formation of stress granules is activated by heat shock, starvation, and dietary restriction in *Caenorhabditis elegans*. (a) The formation of stress granules was visualized by fluorescence microscopy in L3 stage *tiar‐1::gfp* transgenic animals. Worms were treated with or without heat shock 37°C for 15 min. (b) *tiar‐1::gfp* animals were treated with L4440 or *gtbp‐1* RNAi from eggs. Intestinal TIAR‐1::GFP foci under normal conditions or heat shock 37°C for 15 min was observed by fluorescence microscopy. (c) L3 stage *tiar‐1::gfp* transgenic animals were treated with ad libitum conditions or 2‐hr starvation. (d) The formation of TIAR‐1::GFP foci in wild‐type animals and *eat‐2* mutants. Right panels, quantitative analysis of stress granules for (a–d) Each data point represents the number of stress granules per intestinal cell obtained from one image. Data were analyzed by Student's *t* test or two‐way ANOVA. Levels of significance are shown as ***p < .001; ****p < .0001. Scale bar: 20 µm

The transgenic GFP fusion proteins of TIAR‐1 have previously been shown to retain most wild‐type TIAR‐1 activity in vivo (Huelgas‐Morales et al., 2016) and are used as SG markers to track SG dynamics in worms (Lechler et al., 2017; Rousakis et al., 2014). Thus, we first used the TIAR‐1::GFP knock‐in strain to monitor the SG assembly and validate the system under acute heat shock. We found that the formation of TIAR1::GFP granules under acute heat shock was mainly found in the intestine, but not in other tissues. Through time‐lapse analysis, we found that TIAR‐1::GFP foci were generated in the intestinal cells within 15 min after a temperature shift from 20 to 37°C (Figure 1a). Furthermore, the heat‐induced formation of TIAR‐1::GFP foci could be observed from larval to adult stages of worms (Figure S1a). To minimize background autofluorescence in the intestine, we consequently performed most of our SG experiments in L3 to L4 stages. The TIAR‐1::GFP foci induced by acute heat shock in the intestine demonstrated several distinguishing characteristics of SGs. First, TIAR‐1 granules are dynamic. Less TIAR‐1 granules induced by heat shock were visible after 8 hr of 20°C recovery (Figure S1b). Previous studies have shown that mammalian G3BP is one of the SG core proteins and is essential for SG formation. Thus, the formation of TIAR‐1 granules should be affected by G3BP depletion if the heat‐induced TIAR‐1::GFP foci we observed are stress granules. Indeed, when we knocked down GTBP‐1, the worm homolog of G3BP, heat shock‐induced TIAR‐1::GFP foci were significantly reduced (Figure 1b), suggesting that *gtbp‐1* is required in the generation of TIAR‐1 granules. Furthermore, using a mutant strain harboring GTBP‐1::GFP, we found that acute heat shock could also trigger the formation of GTBP‐1::GFP foci in worms and that this GTBP‐1 granule formation is significantly inhibited by *tiar‐1* knockdown (Figure S1d). The functionality of the GFP‐tagged GTBP‐1 in vivo was confirmed by an oxidative stress resistance assay (Figure S1c). Interestingly, unlike TIAR‐1::GFP foci, GTBP‐1::GFP foci under heat shock were found not only in the intestine but also in other tissues (Figure S1c), suggesting that the composition of stress granules might vary in different tissues. Together, our data indicate that both GTBP‐1/G3BP and TIAR‐1/TIA‐1 are key regulators of stress granule formation in *C. elegans,* as observed in other systems.

Recent studies have shown that nutrient deprivation could activate SG formation in cells, yeast, and the single‐celled organism, *T. crucei* (Kafkova et al., 2018). To investigate whether starvation or dietary restriction could induce stress granule formation in *C. elegans*, TIAR‐1::GFP knock‐in animals at L3 stages were transferred to foodless plates for two hours and SG formation was observed. We found that TIAR::GFP foci in the intestine were strongly induced after two hours of starvation (Figure 1c). Starvation‐induced TIAR‐1 granules can be observed in larval stages and in adults up to Day 4 (Figure S2a). We also found that starvation could trigger the formation of GTBP‐1 granules in the intestine as well (Figure S2b). RNAi knockdown of *gtbp‐1* markedly reduced TIAR‐1 granule formation in response to starvation (Figure S2c). Consistent with the dynamic feature of SGs, intestinal TIAR‐1::GFP granules disappeared after a 6‐hr recovery period from starvation (Figure S2d).

Our results indicate that SG formation in the intestine may be triggered by dietary changes in *C. elegans*. Thus, we next tested whether dietary restriction (DR) could also induce intestinal SG formation. To address this, we introduced TIAR‐1::GFP transgene into *eat‐2* mutants, a genetic model for dietary restriction. We found that elevated TIAR‐1 granules appeared in the intestine of *eat‐2* mutants even under normal feeding conditions (Figure 1d). This intestinal TIAR‐1 granules could be detected in adult worms up to Day 4 of adulthood (Figure S3a). To further confirm that DR could trigger SG formation, we applied direct dietary restriction (sDR) on TIAR‐1::GFP reporter animals. The animals under sDR condition also demonstrated an increased number of SGs in the intestine (Figure S3b). Moreover, sDR‐triggered SG formation can be observed in both larval and adult worms (Figure S3b). Similar to the stress granules induced by heat shock or starvation, the number of the sDR‐induced TIAR‐1 granules decreased after 6 hr of recovery (Figure S3c).

### 2.2 Stress granule formation induced by acute heat stress and starvation requires HSF‐1 in * Caenorhabditis elegans*

**FIGURE 2:**
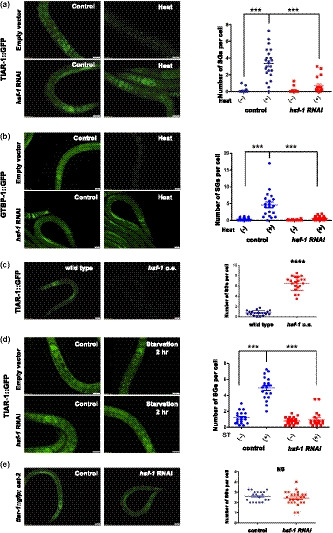
HSF‐1 is required for heat shock and starvation‐induced stress granule formation in *Caenorhabditis elegans*. (a) *tiar‐1::gfp* transgenic animals were treated with L4440 or *hsf‐1* RNAi from eggs. TIAR‐1::GFP foci in the intestinal cells was observed under normal conditions or heat stress. (b) *gtbp‐1::gfp* transgenic animals were treated with L4440 or *hsf‐1* RNAi from eggs. GTBP‐1::GFP foci in the intestinal cells was analyzed with or without heat stress. (c) TIAR‐1::GFP in wild‐type and *hsf‐1* overexpressing mutant backgrounds. (d) *tiar‐1::gfp* transgenic animals were treated with L4440 or *hsf‐1* RNAi from eggs. TIAR‐1::GFP foci in the intestinal cells were observed under ad libitum conditions or 2‐hr starvation. (e) *eat‐2* mutants carrying TIAR‐1::GFP were treated with L4440 or *hsf‐1* RNAi from eggs. Right panels, quantitative analysis of stress granules for (a–e). Each data point represents the number of stress granules per intestinal cell obtained from one image. Data were analyzed by Student's *t* test or two‐way ANOVA. Levels of significance are shown as ***p < .001. NS, not significant. Scale bar: 20 µm

Heat shock factor 1 (HSF1) is known to be the master regulator of heat shock responses in eukaryotes. In *C. elegans,* HSF‐1 is involved not only in the regulation of stress responses but also in other physiological functions, such as metabolism and longevity. Since heat shock is one of the most consistent stressors to induce SG formation in various model systems, we tested whether HSF‐1 plays a role in the regulation of SG generation in worms. To examine this, we fed the TIAR‐1::GFP or GTBP‐1::GFP reporter animals with *hsf‐1* RNAi bacteria. We found that TIAR‐1 granule formation induced by heat shock is significantly suppressed by RNAi knockdown of *hsf‐1* (Figure 2a). Similarly, HSF‐1 depletion abolished the formation of GTBP‐1 granules in response to heat shock (Figure 2b), whereas overexpression of HSF‐1 promotes TIAR‐1 granule formation (Figure 2c), suggesting that the level of HSF‐1 activity is critical for SG formation.

We next investigated whether the starvation or DR‐induced SG formation is also dependent on HSF‐1. We found that RNAi knockdown of HSF‐1 in TIAR‐1::GFP worms markedly reduced the levels of stress granules induced by 2‐hr starvation treatments (Figure 2d). On the other hand, RNAi knockdown of HSF‐1 did not affect SG formation in *eat‐2* mutants (Figure 2e). Together, our findings suggest that HSF‐1 is involved in the SG formation induced by heat and starvation, but not DR.

### 2.3 Stress granule formation induced by dietary changes, but not heat stress, requires AMPK in * Caenorhabditis elegans*

**FIGURE 3:**
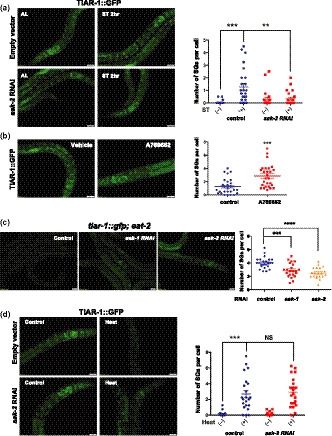
Starvation and diet restriction induce stress granule formation via AMPK in *Caenorhabditis elegans*. (a) L3 stage *tiar‐1::gfp* transgenic animals grown on control or *aak‐2* RNAi bacteria were treated with ad libitum conditions or 2‐hr starvation. (b) L3 stage *tiar‐1::gfp* transgenic animals were treated with vehicle or 0.1 mM A769662 for 24 hr. (c) The formation of TIAR‐1::GFP granules in *eat‐2* mutants fed with bacteria containing control vector, *aak‐1*, or *aak‐2* RNAi plasmids. (d) *tiar‐1::gfp* transgenic animals grown on control or *aak‐2* RNAi bacteria were treated with or without heat stress. Right panels, quantitative analysis of stress granules for (a–d). Each data point represents the number of stress granules per intestinal cell obtained from one image. Data were analyzed by Student's *t* test, one‐way or two‐way ANOVA. Levels of significance are shown as **p < .01; ***p < .001; ****p < .0001. NS, not significant. Scale bar: 20 µm

Previous studies have suggested that the regulation of SG formation might be stress‐specific. For instance, heat‐induced SG assembly requires the phosphorylation of the α‐subunit of the eukaryotic initiation factor 2 (eIF2α), whereas the formation of SGs induced by hyperosmotic stress is independent of eIF2α phosphorylation (Aulas et al., 2017). Thus, we further investigated the signaling pathways that may promote SG formation in response to starvation or dietary restriction. AMP‐activated protein kinase (AMPK) is one of the key regulators for starvation stress responses. Previously, it has been reported that activation of AMPK by a pharmacological activator A769662 can promote SG formation in cultured cells (Mahboubi, Koromilas, & Stochaj, 2016). To examine whether AMPK signaling is involved in the SG formation triggered by starvation*,* AMPK activity was depleted in TIAR‐1::GFP animals by *aak‐2* RNAi. We found that inhibition of *aak‐2* activity significantly suppressed the SG formation upon 2 hr of starvation (Figure 3a). To further confirm that elevated AMPK activity could facilitate the formation of SGs, we treated the TIAR‐1::GFP worms with AMPK activator A769662. We found that SG formation was greatly enhanced after one day of 0.1 mM A769662 treatment (Figure 3b). Together, our data suggest that AMPK is required for starvation‐induced SG formation.

Next, we tested whether AMPK plays a role in the SG formation induced by DR or heat stress. Interestingly, we found that RNAi knockdown of *aak‐1* or *aak‐*2 partially suppressed the SG formation in *eat‐2* mutants (Figure 3c), while SG formation induced by heat stress was not affected by the depletion of *aak‐1* or *aak‐2* (Figure 3d and Figure S4a). These findings again indicate that heat shock and starvation might promote stress granule formation through distinct pathways in *C. elegans*.

### 2.4 AMPK regulates stress granule formation via the eEF2K‐eEF2 pathway

**FIGURE 4:**
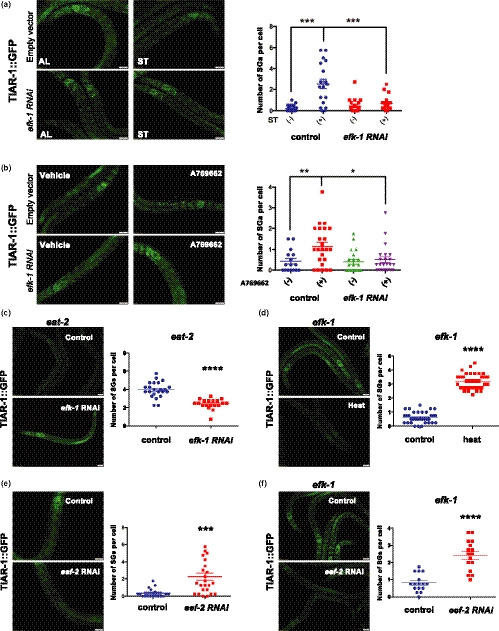
*efk‐1/* eEF2K is the downstream regulator of AMPK‐dependent SG formation. (a) *tiar‐1::gfp* transgenic animals subjected to control or *efk‐1* RNAi bacteria were treated with ad libitum conditions or 2‐hr starvation. (b) *tiar‐1::gfp* transgenic animals fed with control vector or *efk‐1* RNAi bacteria were treated with vehicle or 0.1 mM A769662 for 24 hr. (c) *eat‐2* mutants carrying TIAR‐1::GFP were treated with L4440 or *efk‐1* RNAi from eggs. (d) The formation of TIAR‐1::GFP granules in *efk‐2* mutants was observed under normal conditions or heat stress. (e) The formation of TIAR‐1::GFP foci in worms fed with bacteria containing control vector or *eef‐2* RNAi plasmids. (f) *efk‐2* mutants carrying TIAR‐1::GFP were treated with L4440 or *eef‐2* RNAi from eggs. Right panels, quantitative analysis of stress granules for (a–f). Each data point represents the number of stress granules per intestinal cell obtained from one image. Data were analyzed by Student's *t* test or two‐way ANOVA. Levels of significance are shown as *p < .05; **p < .01; ***p < .001; ****p < .0001. Scale bar: 20 µm

As stress granule assembly is often coupled with translation repression by sequestering mRNAs, we next turned to AMPK downstream effectors that could affect protein synthesis in the regulation of SG formation. Several pieces of evidence indicate that AMPK might affect protein synthesis via mTOR or eEF2K pathways in cultured cells (Johanns et al., 2017). However, since it has been reported that TOR activity is required for the SG formation (Jevtov et al., 2015), it is unlikely AMPK mediates SG assembly by inhibiting the TOR pathway. Thus, we examined whether eEF2K signaling is involved in the formation of starvation‐induced SGs. To do so, we knocked down *efk‐1*, worm homolog of eEF2K, in starved TIAR‐1::GFP worms. Our results indicated that the depletion of *efk‐1* by RNAi strongly suppressed the SG formation in response to starvation (Figure 4a). To confirm that EFK‐1 acts downstream of AMPK to regulate SG formation, 0.1 mM AMPK activator A769662 was introduced to TIAR‐1::GFP animals fed with control or *efk‐1* RNAi bacteria. We found that RNAi depletion of *efk‐1* diminished SG formation triggered by AMPK activation (Figure 4b). Moreover, RNAi knockdown of *efk‐1* inhibits the generation of TIAR‐1 granules in *eat‐2* mutants as well (Figure 4c). Similar to AMPK, deletion of *efk‐1* does not affect the heat‐induced SG formation in worms (Figure 4d).

The most well‐characterized target of eEF2K in cells is eEF2, the eukaryotic elongation factor 2. Upon activation by upstream kinases, eEF2K phosphorylates and thereby inactivates eEF2, leading to translation inhibition. However, whether the AMPK‐eEF2K‐eEF2 signaling is involved in the induction of SG assembly has not yet been studied. If eEF2 is required in AMPK‐eEF2K‐mediated SG formation, inactivation of *eef‐2*, the worm homolog of eEF2, should be able to induce SG formation. Indeed, we found that knockdown of *eef‐2* markedly induced SG formation even in the absence of stressors (Figure 4e). Moreover, down‐regulation of *eef‐2* by RNAi promotes SG formation in *efk‐1* mutants (Figure 4f). Together, our data suggest that EEF‐2/eEF2 might act downstream of eEF2K to regulate diet‐induced SG formation.

### 2.5 AAK‐1‐mediated stress granules formation is required for DR‐induced, but not IIS signaling‐mediated lifespan extension

**FIGURE 5:**
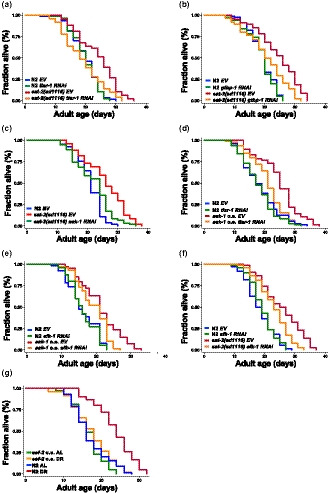
SG formation and AMPK‐eEF2K pathway are required for DR‐induced longevity in *Caenorhabditis elegans*. Lifespan analysis of (a) wild‐type N2 animals (blue and green) and *eat‐2* mutants (red and orange) treated with empty vector or *tiar‐1* RNAi bacteria from L4 stage. (b) wild‐type N2 animals (blue and green) and *eat‐2* mutants (red and orange) fed with empty vector or *gtbp‐1* RNAi bacteria from L4 stage. (c) wild‐type N2 animals (blue) and *eat‐2* mutants (red and green) grown on empty vector or *aak‐1* RNAi bacteria from L4 stage. (d) of wild‐type N2 (blue and green) and *aak‐1* overexpressing mutants (red and orange) worms grown on empty vector or *tiar‐1* RNAi bacteria. (e) wild‐type N2 (blue and green) and *aak‐1* overexpressing (red and orange) worms grown on empty vector or *efk‐1* RNAi bacteria from L4 stage. (f) wild‐type N2 (blue and green) and *eat‐2* mutants (red and orange) grown on empty vector or *efk‐1* RNAi bacteria from L4 stage. (g) wild‐type N2 (blue and red) and *eef‐2* overexpressing mutants (green and orange) grown on 5 × 10^10^ (AL) or 1 × 10^8^ (DR) cells/ml of bacteria from Day 1 adult stage. Additional lifespan replicates and statistical analyses are included in Table S1

The protective functions of TIAR‐1 in the reproduction system of *C. elegans* have been previously reported (Huelgas‐Morales et al., 2016). Similarly, *gtbp‐1* deletion mutants are known to be more sensitive to heat and oxidative stresses and have short lifespan (Huang, Wu, Wang, & Zhang, 2018). These observations suggest that SG formation may be one of the inducible mechanisms for confronting stresses in animals. Since it is known that stress responses and longevity regulation are often connected and that DR results in a robust lifespan extension, we then asked whether SG formation plays a role in DR‐induced longevity. As mentioned above, elevated levels of SGs were observed in both direct sDR animals and *eat‐2* mutants (Figure 1d and Figure S2d). To investigate whether SG formation is required for the longevity phenotype in DR animals, we knocked down *tiar‐1* by RNAi in *eat‐2* mutants during only adulthood and found that their lifespan extension was significantly suppressed (Figure 5a). Similarly, adult‐only *gtbp‐1* RNAi significantly suppresses the lifespan extension of *eat‐2* mutations (Figure 5b), suggesting that SG formation might be required for DR‐induced longevity.

While AMPK has been implicated in longevity regulation in several different animal model, its role in DR‐induced longevity in worms varies, depending on the DR regiments utilized. While it has been reported that AMPK activity was required for lifespan extension in sDR (Greer & Brunet, 2009), inactivation of *aak‐2* does not shorten the lifespan of *eat‐2* mutants (Curtis, O'Connor, & DiStefano, 2006). It is noteworthy that there are two α isoforms of AMP‐activated protein kinase in *C. elegans,* AAK‐1 and AAK‐2*.* However, little research has been done on AAK‐1. Given its role in diet‐related SG formation, we tested whether *aak‐1* also plays a role in DR‐induced longevity. Interestingly, we found that adult‐only *aak‐1* RNAi markedly suppresses the lifespan of *eat‐2* animals, whereas whole‐life *aak‐1* RNAi does not (Figure 5c). Moreover, *aak‐1* overexpression significantly increases longevity in worms by 20 to 35% (Figure 5d,e). Since we have demonstrated in this study that SG formation induced by DR requires AMPK‐eEF2K‐eEF2 signaling, we next tested whether SG formation is required for the lifespan extension in *aak‐1* overexpressing animals. We found that knockdown of *tiar‐1* during adulthood markedly shorten the lifespan of long‐lived *aak‐1* overexpressing animals (Figure 5e). Furthermore, depletion of *efk‐1/eEF2K* by RNAi during only adulthood shortens the lifespans of both *aak‐1* overexpressing animals and *eat‐2* mutants (Figure 5e,f), suggesting that *efk‐1* acts downstream of *aak‐1* to regulate the longevity response to dietary restriction. We also found that adult‐only knockdown of *eef‐2* significantly extends the lifespan of wild‐type animals, but not *eat‐2* mutants (Figure S4b). To confirm the role of *eef‐2* in DR‐induced longevity, lifespan analysis was performed on *eef‐2* overexpressing mutants with direct sDR (Figure 5f). Our results indicate that overexpression of *eef‐2* significantly suppresses the longevity effect of DR. Taken together, inhibition of SG formation or reducing AAK‐1‐eEF2K‐eEF2 signaling could suppress longevity response of dietary restriction in *C. elegans*.

**FIGURE 6:**
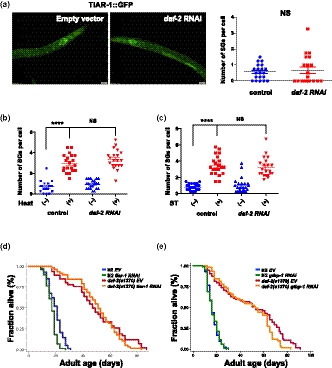
SG formation is not required for Insulin/IGF‐1‐like signaling (IIS)‐mediated longevity. (a) The formation of TIAR‐1::GFP foci in wild‐type animals fed with bacteria containing control vector or *daf‐2* RNAi plasmids. Right panel, quantitative analysis of stress granules. SG quantification in *tiar‐1::gfp* transgenic animals treated with L4440 or *daf‐2* RNAi from eggs under (b) normal conditions or heat stress (c) ad libitum or starvation. Data were analyzed by Student's *t* test or two‐way ANOVA. Levels of significance are shown as ****p < .0001. NS, not significant. Scale bar: 20 µm. (d) Lifespan analysis of wild‐type N2 (blue and green) and *daf‐2* mutant (red and orange) worms grown on empty vector or *tiar‐1* RNAi bacteria. (e) Lifespan analysis of wild‐type N2 (blue and green) and *daf‐2* mutant (red and orange) worms grown on empty vector or *gtbp‐1* RNAi bacteria from L4 stage. Additional lifespan replicates and statistical analyses are included in Table S1

We then tested whether SG formation is involved in other longevity regulatory pathways, such as the Insulin/IGF1‐like signaling (IIS). To test this, we first examined whether a reduction of IIS could trigger SG formation. Surprisingly, the reduction of IIS signaling could not induce TIAR‐1 or GTBP‐1 granule formation (Figure 6a and Figure S5a). It has been reported that reduced IIS signaling prevents age‐dependent aggregation of stress granule proteins (Lechler et al., 2017). Hence, we would like to know whether knockdown of *daf‐2* could inhibit heat or diet‐induced SG formation in young adults. We found that SG formations under acute heat shock or 2‐hr starvation were not affected by *daf‐2* RNAi (Figure 6b,c). Moreover, the number of TIAR‐1 puncta in *eat‐2* mutants was unchanged by *daf‐2* RNAi (Figure S5b). These data indicate that the IIS signaling is not required for the SG formation upon heat or diet stress. However, it is possible that reduced IIS signaling might delay the age‐dependent formation of solid SG protein aggregation via increasing proteostasis in the cells. We then knocked down TIAR‐1 or GTBP‐1 in *daf‐2* mutants and performed lifespan analysis. Consistently, the lifespan of *daf‐2* mutants was not affected by *tiar‐1* or *gtbp‐1* RNAi (Figure 6d,e). Together, our data indicate that SG formation is required for certain but not all the longevity pathways to control lifespan.

## 3 DISCUSSION

As our results have demonstrated, in addition to acute environmental stressors (e.g., heat), stress granule formation in the intestine of *C. elegans* could be triggered by dietary changes (e.g., starvation or dietary restriction). Consistent with studies done in mammalian cells, TIAR‐1/TIA1 and GTBP‐1/G3BP are present in the stress granules and required for the SG formation in *C. elegans*. Both TIAR‐1 and GTBP‐1 are ubiquitously expressed in worms. However, TIAR‐1 granules exist mainly in the intestine, whereas GTBP‐1 granules could be found in most of the tissues. The differences in the tissue distribution of these two proteins have supported the idea that SGs may have different subtypes (Panas, Ivanov, & Anderson, 2016; Reineke, Cheema, Dubrulle, & Neilson, 2018). Interestingly, we found that *tiar‐1* knockdown significantly inhibited the heat‐induced GTBP‐1 granule formation in all tissues, while TIAR‐1 foci could mostly be found in the intestine. These observations suggest that TIAR‐1 functions not only as a component of a certain subtype of SG but also as a regulator in the generation of other subtypes of SG.

Our results have also shown that HSF‐1 plays an important role in the regulation of SG formation in response to heat‐induced SG formation. It is known that HSF‐1 could be activated by heat stress or reduced IIS signaling via disrupting the HSF‐1/HSPs or DHIC complexes, respectively (Chiang, Ching, Lee, Mousigian, & Hsu, 2012; Morimoto, 2002). However, no SG formation was observed when we knocked down either *daf‐2* RNAi (Figure 6) or *hsb‐1* RNAi (data not shown). And reduced IIS signaling could not prevent SG formation induced by heat or starvation (Figure 6b,c), suggesting that the IIS‐DHIC pathway is not required for SG assembly. Previous proteomic studies of stress granules in yeast and mammalian cells have identified several heat shock proteins, such as HSP70 and HSP40, as main core proteins in the SGs (Jain et al., 2016). Moreover, previous studies have shown that HSP90 is involved in the formation of stress granules (Matsumoto et al., 2011). Thus, acute heat shock might trigger the dissociation of the HSF‐1/HSPs complex. The released HSPs would then facilitate the SG assembly. Meanwhile, the activated HSF‐1 would further increase the expression of HSPs required in SG formation.

SG formation induced by nutrient deprivation has only been observed in single‐cell systems. Here, we have demonstrated that both starvation and dietary restriction can trigger SG formation at the organismal level. To elucidate the signaling pathways that mediate the SG formation in response to starvation or DR, we focused on one of the main nutrient‐sensing pathways, the AMPK signaling. The activation of AMPK (Mahboubi et al., 2016) has been reported to induce SG formation in cultured cells. In *C. elegans*, we found that stress granules induced by starvation and DR were both mediated by AMPK. We also identified that eEF2K and eEF2 are the downstream mediators of AMPK signals that control the generation of stress granules in *C. elegans*. Previously, *Leprivier et al.* have shown that *efk‐1/eEF2K* may mediate the stress response to nutrient deprivation in both cultured cells and *C. elegans* (Leprivier et al., 2013). The authors also suggested that eEF2K inhibits translation elongation to increase stress resistance. Here, we found that the inactivation of *eef‐2/eEF2* promotes SG formation, suggesting that eEF2 might serve as a negative regulator of SG formation. Moreover, eEF2 has been reported as a regulator for virus‐induced SG assembly in cell culture (Valiente‐Echeverria et al., 2014). Therefore, since eEF2 and eEF2K are involved in both SG formation and protein translation, dietary changes (i.e., starvation and DR) may suppress protein synthesis and promotes SG formation via an integrated mechanism in which the eEF2K‐eEF2 axis of AMPK pathway is activated.

In previous studies, the involvement of AMPK, particularly the AAK‐2 isoform, in DR‐induced longevity remains regimen‐dependent (Greer & Brunet, 2009). Here, our data suggest that AAK‐1 is required for the lifespan extension of *eat‐2* DR animals, which was previously found to be AAK‐2‐independent. Our epistasis analyses also suggest that the SG formation mediated by the AAK‐1‐eEF2K‐eEF2 axis might contribute to longevity phenotype in DR animals. Thus, the role of AMPK (i.e., AAK‐1 and AAK‐2) signaling in DR‐induced longevity may be broader than previously thought. As aging is often linked to accumulations of damaged proteins, maintaining protein homeostasis (or proteostasis) is critical for longevity regulation (Higuchi‐Sanabria, Frankino, Paul, Tronnes, & Dillin, 2018). Here, our findings suggest that DR may activate the AMPK‐eE2K axis to regulate proteostasis and consequently the rate of aging via inhibiting translation and elevating SG formation.

Interestingly, while it has been shown that protein translation is also reduced in *daf‐2* mutants (Depuydt, Shanmugam, Rasulova, Dhondt, & Braeckman, 2016), we did not observe spontaneous SG formation in *daf‐2* mutants (Figure 6a and Figure S5a). Furthermore, the longevity phenotype of *daf‐2* mutants was not affected by *tiar‐1* or *gtbp‐1* RNAi knockdown (Figure 6d,e). Thus, unlike the DR‐induced longevity, elevated SG formation appears to be dispensable for the IIS pathways to extend lifespan in *C. elegans*.

Accumulation of stress granule‐like RBPs containing TDP‐43, FUS/TLS, or ataxin‐2 is a key feature of several neurodegenerative diseases (Harrison & Shorter, 2017). Since some stress granule markers have been found in those RBP aggregates (Li, King, Shorter, & Gitler, 2013; Vanderweyde et al., 2016), the accumulation of pathological RBP aggregates in patients might be caused by dysfunction of SG regulation. Thus, further understanding the molecular mechanisms by which SG dynamics are regulated in young and old animals would be helpful for the identification of new therapeutic targets combating age‐related neurodegenerative diseases.

## 4 EXPERIMENTAL PROCEDURES

### 4.1 Strains

N2: wild‐type
CB1370: *daf‐2(e1370)III*
DA1116: *eat‐2(ad1116)II*
DG3922: *tiar‐1(tn1545[tiar‐1::gfp::tev::s])II.*
EQ1136: *iqEx225[aak‐1p::aak‐1::sl2::mCherry + rol‐6]*
EQ1336: *eat‐2(ad1116)II; tiar‐1(tn1545[tiar‐1::gfp::tev::s])II*
JH3176: *gtbp‐1(ax2029)IV*
JH3199: *gtbp‐1(ax2055[gtbp‐1::gfp]) IV*
EQ1553: *iqEx313[eef‐2p::eef‐2::gfp + rol‐6]*
EQ1555: *efk‐1(ok3609)III; tiar‐1(tn1545[tiar‐1::gfp::tev::s])II*
EQ1584: *tiar‐1(tn1545[tiar‐1::s::tev::GFP]) II; iqIs37[hsf‐1p::myc‐hsf‐1 + rol‐6]*

DA1116, CB1370, DG3922, JH3199, and wild‐type *Caenorhabditis elegans* (N2) strains were obtained from the Caenorhabditis Genetic Center. All strains were maintained under standard culturing condition.

### 4.2 RNA‐interference (RNAi) clones

The identity of all RNAi clones was verified by sequencing the inserts using M13‐forward primer. The *tiar‐1* RNAi clone was home‐made. All other clones were from Julie Ahringer's RNAi library. HT115 bacteria transformed with RNAi vectors expressing dsRNA of the genes of interest were grown at 37°C in LB with 10 μg/ml tetracycline and 50 μg/ml carbenicillin and then seeded onto NG‐carbenicillin plates containing 1 mM IPTG.

### 4.3 Induction of acute heat shock and starvation

Synchronized worms were loaded onto agar pad and then subjected to a 37°C heat shock for 15 min on stage‐top Micro Heat Plate (Kitazato Corporation). Fluorescence images of live animals were taken at different time points of heat shock. For the starvation assay, animals at the L3 stage were transferred to NGM plates with or without OP50 bacterial lawn for two hours.

### 4.4 Solid plate dietary restriction

The solid plate DR protocol used in our laboratory was adapted and modified based on the method previously described (Ching & Hsu, 2011). OP50 bacteria were grown to near saturation overnight. The concentration of the overnight bacterial culture was measured before diluting into 1 × 10^8^ cells/ml and 5 × 10^10^ cells/ml (5 × 10^10^ cells/ml was considered to be the ad libitum control). A 200 μl of these solutions was seeded on each 35‐mm plate. The plates were kept in 37°C incubator for 1 hr before UV‐killing for 20 min. Both control and DR plates were prepared and stored at 4°C for no more than two days before being used. The worms were placed on plates containing different concentrations of bacteria starting at Day 1 of adulthood.

### 4.5 Stress granule quantification

Synchronized animals carrying *tiar‐1::gfp* or *gtbp‐1::gfp* grown on an empty vector or different RNAi bacteria were treated with heat, 2‐hr starvation, or 16‐hr solid‐phase dietary restriction. Z‐stack DIC and fluorescence images of the animals were taken via an Olympus BX63 microscope with a UPLFLN 40X semi‐apochromat objective using Olympus Microsuite software. Intestinal cell boundaries were determined by DIC images. To evaluate an increase or decrease in the number of TIAR‐1 or GTBP‐1 granules present in the intestine, TIAR‐1::GFP or GTBP‐1::GFP punctate was quantified by using ImageJ “analyze particles” function. To exclude unwanted particles, circularity was set between 0.5 and 1. The number of stress granules per cell was calculated as (number of puncta)/(number of cell). At least 30 animals (1–4 cells per worm) were scored per experiment.

### 4.6 Lifespan analysis

Lifespan analysis was conducted at 20°C as described previously (Apfeld & Kenyon, 1999; Hsu, Murphy, & Kenyon, 2003; Kenyon, Chang, Gensch, Rudner, & Tab tiang, 1993) unless otherwise stated. Strains were grown at 20°C for at least two generations before the experiments were initiated. A total of 60–90 animals were tested in each experiment. RNAi treatments were carried out by adding synchronized eggs, unless noted otherwise, to plates seeded with the RNAi bacteria of interest. For adulthood RNAi knockdown assay, the eggs were incubated at 20°C until the worms reached L4 larval stage and were transferred to RNAi plates. Worms were moved to fresh RNAi plates every two days until reproduction ceased. Worms were then moved to new plates every 5–7 days for the rest of the lifespan analysis. The viability of the worms was scored every two days. In all experiments, the prefertile period of adulthood was used as *t* = 0 for lifespan analysis. Stata 12 (StataCorp) software was used for statistical analysis to determine the means and percentiles. In all cases, *p* values were calculated using the log‐rank (Mantel–Cox) method.

## CONFLICT OF INTEREST

The authors declare no competing financial interests.

## AUTHOR CONTRIBUTIONS

T‐T. C. conceived the project and designed the experiments. G‐T. Y., L‐T. J., C‐T. K., Y‐C. S., and T‐C. C. performed the experiments and analyzed the data. T‐T. C. and A‐L. H. interpreted the data. T‐T. C. and A‐L. H. wrote and edited the manuscript.

## Supporting information

Figure S1

Figure S2

Figure S3

Figure S4

Figure S5

Table S1

Table S2

Figure Legends

## Data Availability

Raw data are available through Mendeley Data: https://data.mendeley.com/datasets/4xgthfd7mv/draft?a=713cbe00‐2be8‐4539‐ae57‐a7dbeadb8022 (Ching, 2019).
