# Supplementary figures and images for: AMPK‐mediated formation of stress granules is required for dietary restriction‐induced longevity in Caenorhabditis elegans
Source: Aging Cell. 2020 May 20;19(6):e13157. doi: 10.1111/acel.13157 (PMC7294782; doi:10.1111/acel.13157)

# Extended figure S1

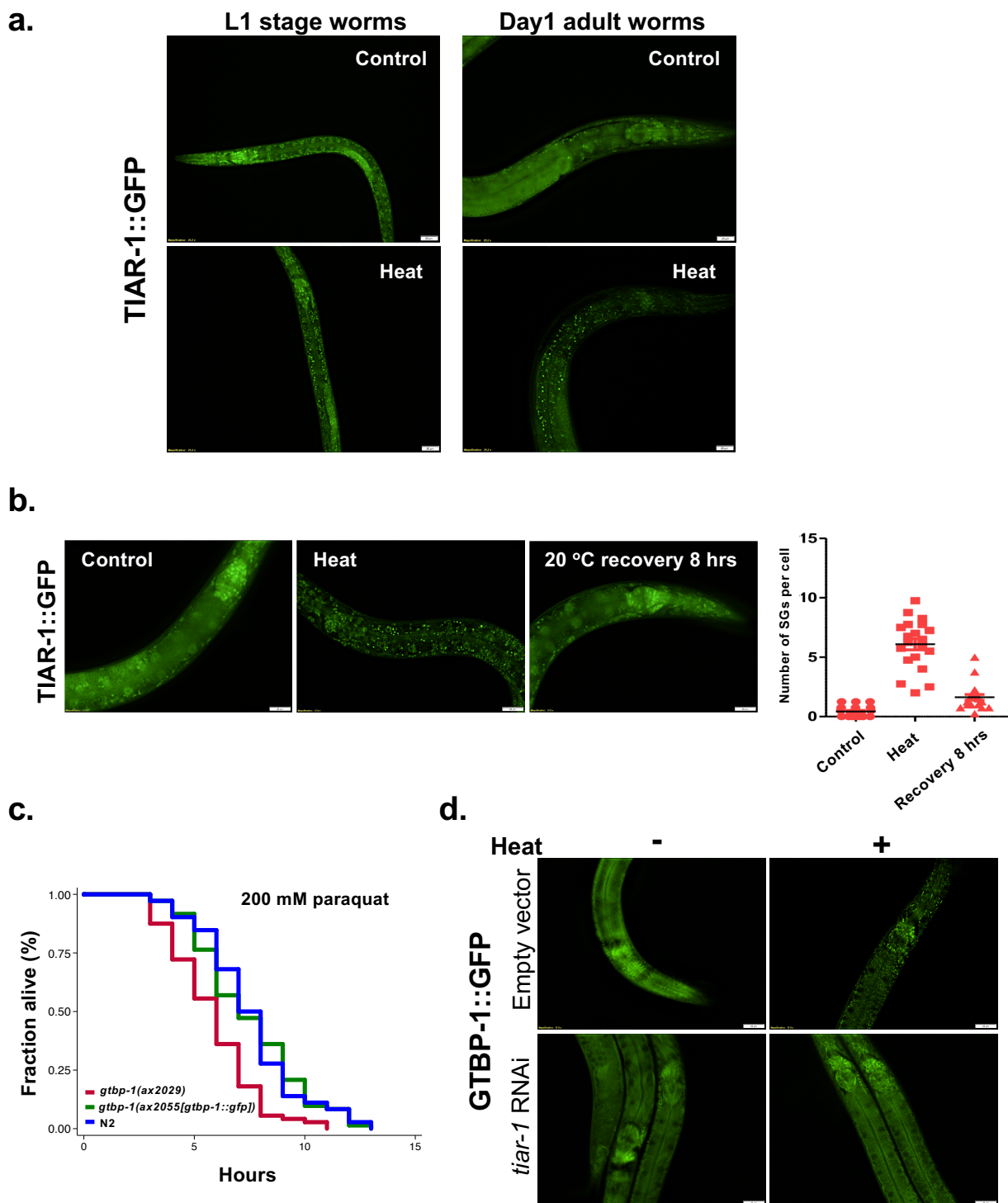

Supplement: Supplementary file 1 — Figure S1 [file ACEL-19-e13157-s001.pdf]

# Extended figure S2

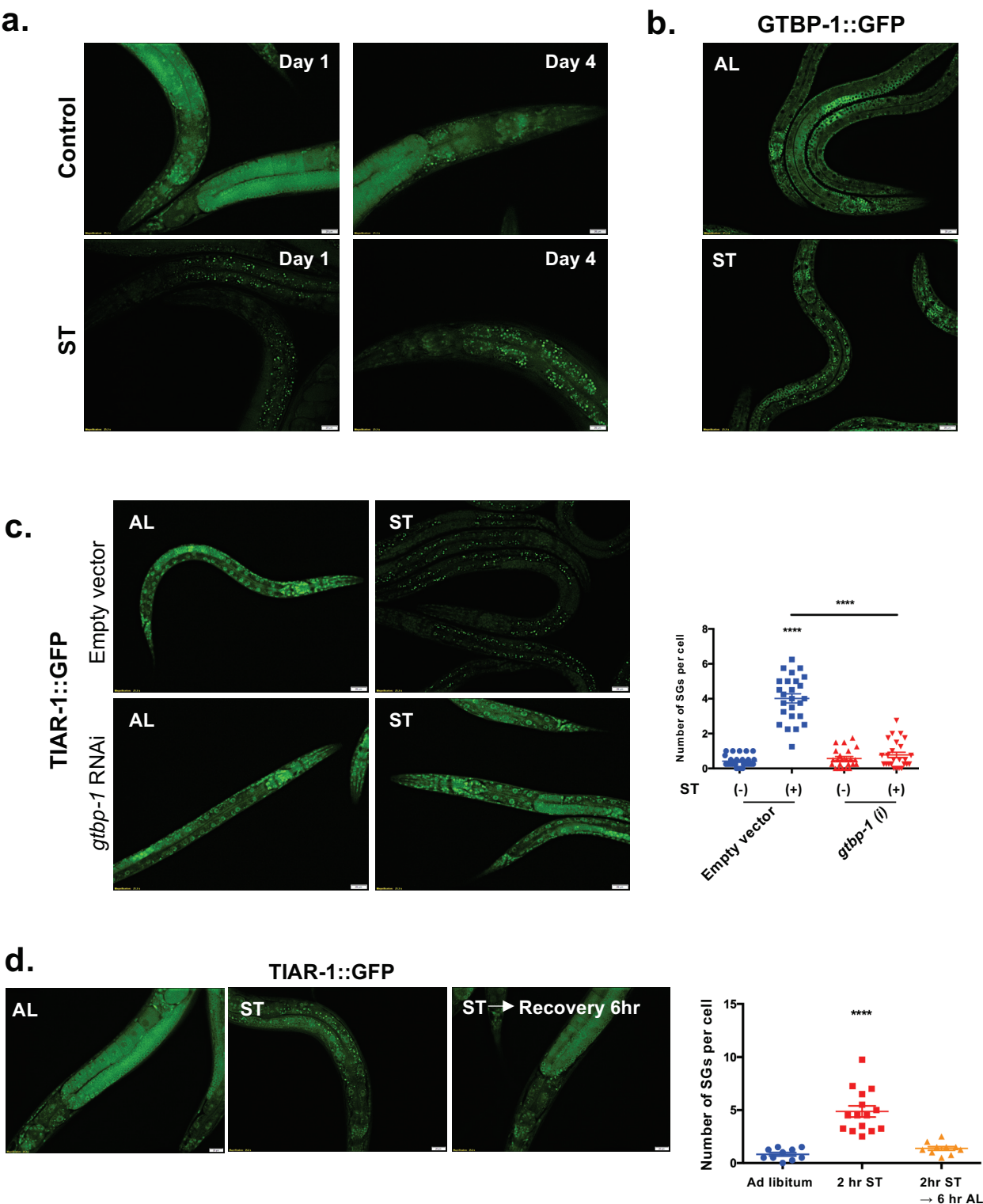

Supplement: Supplementary file 2 — Figure S2 [file ACEL-19-e13157-s002.pdf]

# Extended figure S3

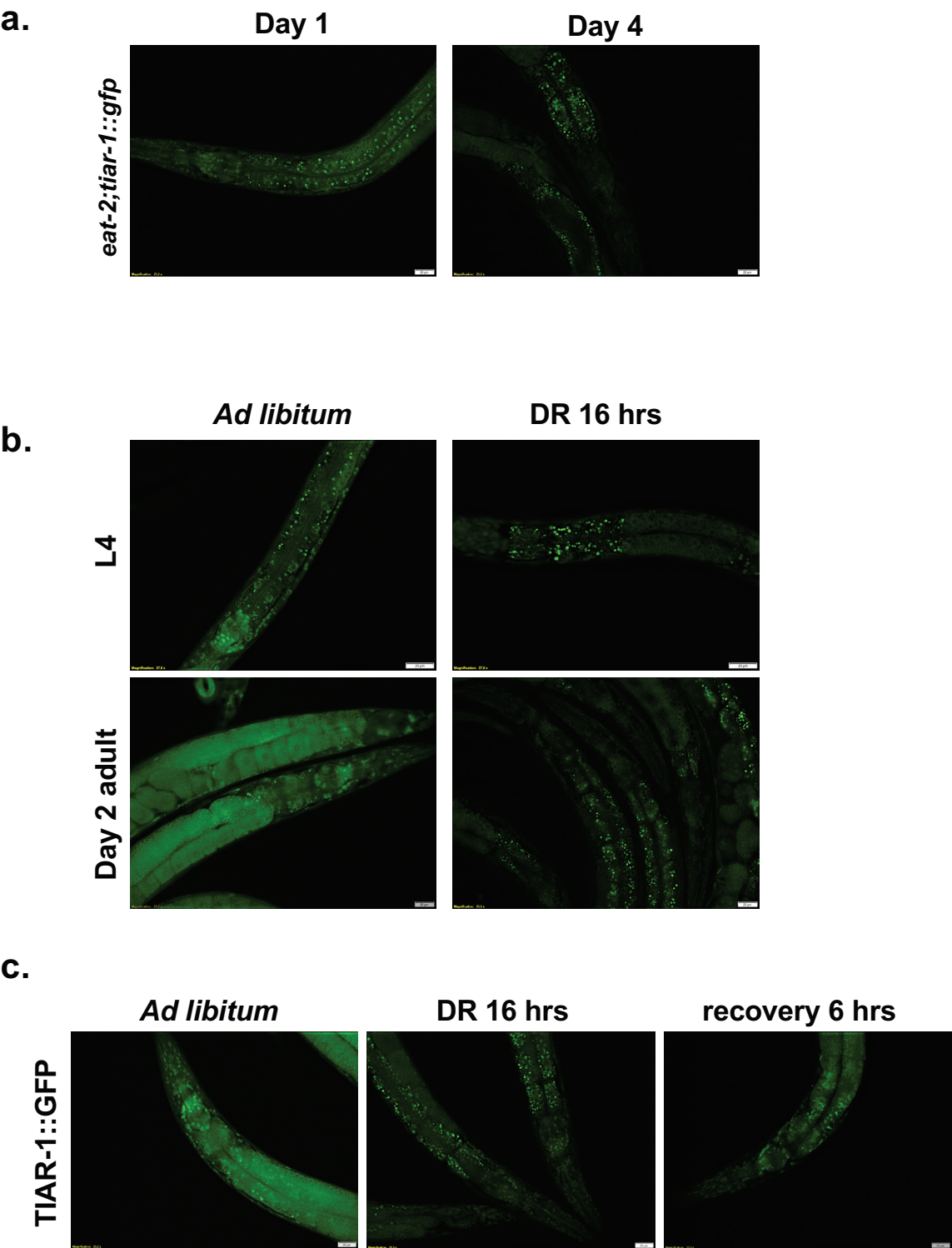

Supplement: Supplementary file 3 — Figure S3 [file ACEL-19-e13157-s003.pdf]

# Extended figure S4

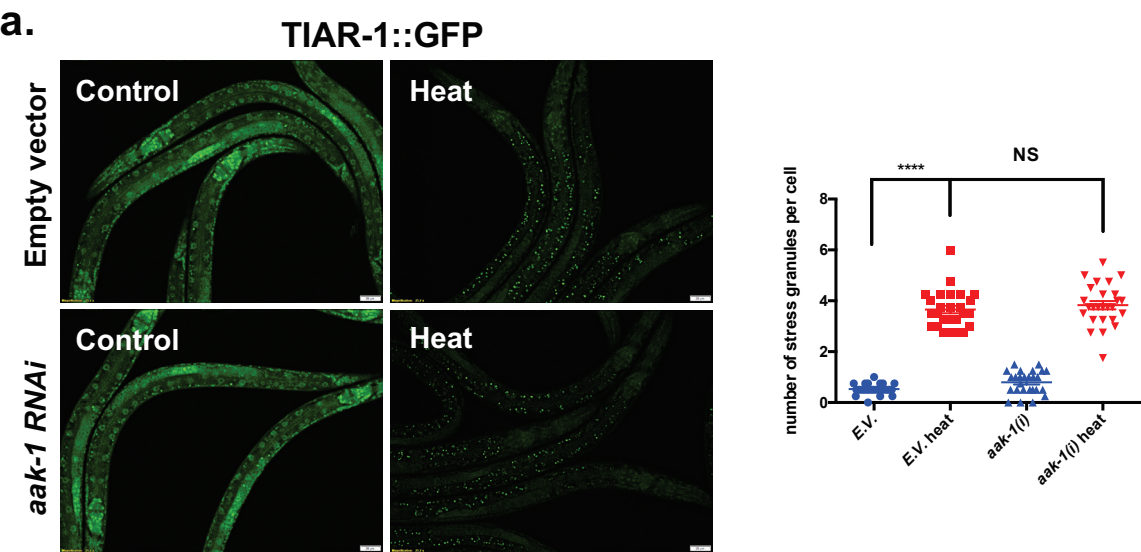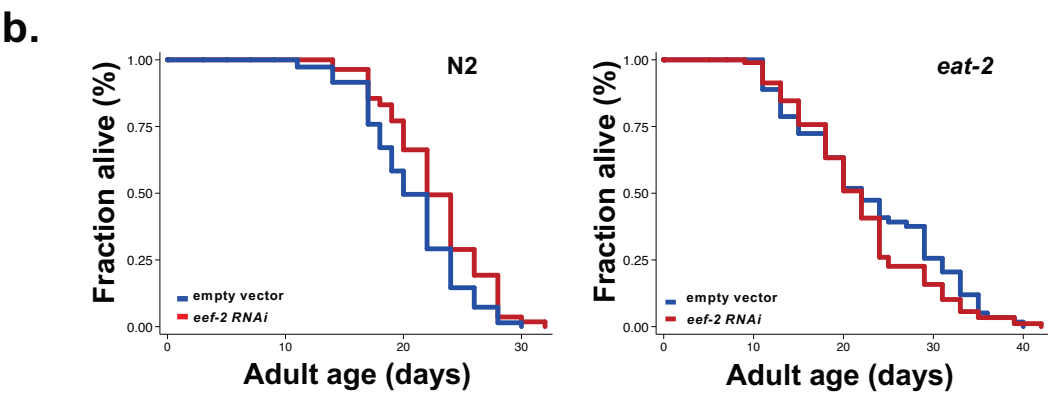

Supplement: Supplementary file 4 — Figure S4 [file ACEL-19-e13157-s004.pdf]

# Extended figure S5

a.

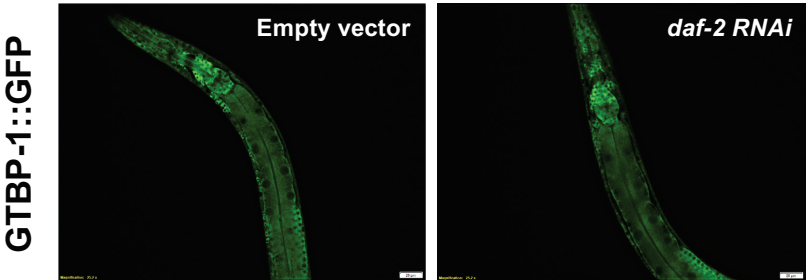

b.

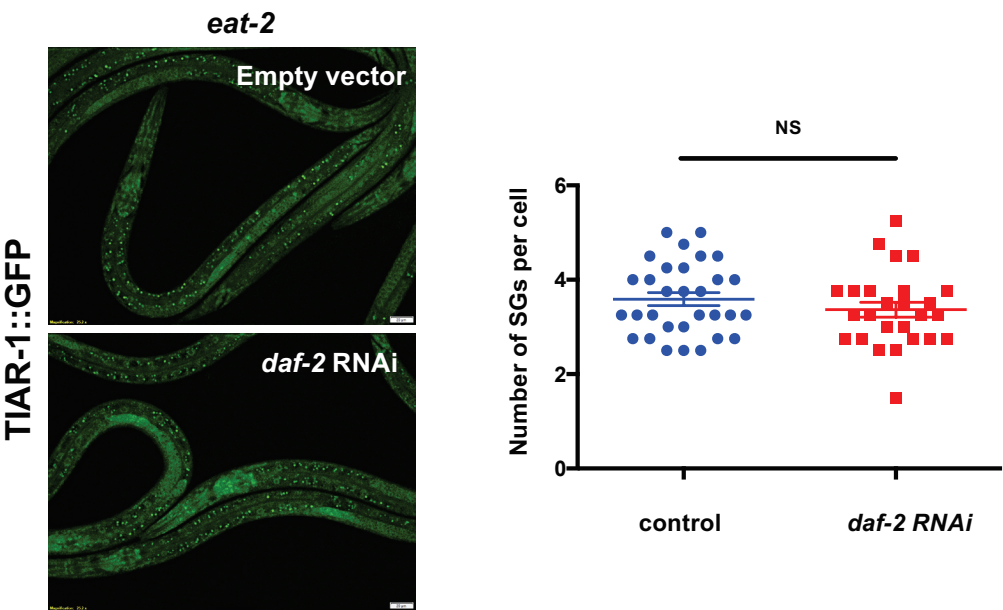

Supplement: Supplementary file 5 — Figure S5 [file ACEL-19-e13157-s005.pdf]
