# Supplementary material for: AMPK‐mediated formation of stress granules is required for dietary restriction‐induced longevity in Caenorhabditis elegans
Source: Aging Cell. 2020 May 20;19(6):e13157. doi: 10.1111/acel.13157 (PMC7294782; doi:10.1111/acel.13157)
Supplement: Supplementary file 6 — Table S1 [file ACEL-19-e13157-s006.pdf]

Extended Data Table 1 | Statistical data for *C. elegans* lifespan experiments

strain name	treatment	mean(days)	*p value*	75%	n	% change (vs N2)	
N2 (WT)	EV	17.68		21	79/84		
N2 (WT)	*tiar-1* RNAi	17.01	0.4437	21	73/84		
eat2	EV	25.88		31	58/84	46.38	
eat2	*tiar-1* RNAi	19.77	< 0.0001	25	62/84	11.82	
N2 (WT)	EV	16.35		20	65/72		
N2 (WT)	*tiar-1* RNAi	15.74	0.5573	20	66/72		
eat2	EV	24.26		30	65/72	48.37	
eat2	*tiar-1* RNAi	19.8	0.0001	22	61/72	18.35	
N2 (WT)	EV (L4)	19.75		22	59/71		**Fig 5a**
N2 (WT)	*tiar-1* RNAi (L4)	19.77	0.9906	22	50/71		
eat2	EV (L4)	23.84		28	51/71	23.44	
eat2	*tiar-1* RNAi (L4)	19.03	0.0004	22	54/72	-5.32	
N2 (WT)	EV (L4)	18.25		21	55/63		
N2 (WT)	*tiar-1* RNAi (L4)	17.69	0.4265	21	61/73		
eat2	EV (L4)	23.39		27	56/82	28.16	
eat2	*tiar-1* RNAi (L4)	21.08	0.0138	25	78/83	15.51	
N2 (WT)	EV (L4)	19.27		22	78/84		**Fig. 5b**
N2 (WT)	*gtbp-1* RNAi (L4)	18.8	0.6443	22	75/84		
eat2	EV (L4)	24.38		30	71/84	26.52	
eat2	*gtbp-1* RNAi (L4)	20.53	0.0008	26	79/84	6.54	
N2 (WT)	EV (L4)	19.75		24	59/71		
N2 (WT)	*gtbp-1* RNAi (L4)	20.55	0.4373	24	65/72		
eat2	EV (L4)	24.38		30	48/70	23.44	
eat2	*gtbp-1* RNAi (L4)	20.62	0.0236	26	45/72	4.41	
N2 (WT)	EV (L4)	20.78		24	56/84		
N2 (WT)	*aak-1* RNAi (L4)	18.99	0.052	21	65/80		
eat-2	EV (L4)	25.78		32	52/84	24.06	
eat-2	*aak-1* RNAi (L4)	21.84	0.0005	26	64/82	5.1	
N2 (WT)	EV (L4)	21.28		24	50/71		**Fig. 5c**
eat-2	EV (L4)	27.43		33	56/75	28.9	
eat-2	*aak-1* RNAi (L4)	24.32	0.0028	29	56/75	14.29	
N2 (WT)	EV (L4)	21.18		24	50/71		
eat-2	EV (L4)	27.36	< 0.0001 (vs N2)	33	54/73	29.17	
eat-2	*aak-1* RNAi (eggs)	26.58	0.5169 (vs *eat-2* EV)	33	49/74	25.5	
eat-2	*aak-1* RNAi (L4)	24.26	0.0037 (vs *eat-2* EV)	29	56/75	14.54	
N2 (WT)		15.76		19	59/72		
aak-1 OE		18.9	0.0005	23	64/72	19.92	
N2 (WT)	EV	18.33		23	72/85		**Fig. 5d**
N2 (WT)	*tiar-1* RNAi	17.87	0.7876	21	72/84		
aak-1 OE	EV	24.71	< 0.0001 (vs N2)	28	76/85	34.8	
aak-1 OE	*tiar-1* RNAi	20.88	0.0001 (vs *aak-1* OE)	25	78/83	13.91	
N2 (WT)	EV	16.05		21	64/84		**Fig. 5e**
N2 (WT)	*efk-1* RNAi (L4)	16.2	0.8307	21	71/84		
aak-1 OE	EV	21.28	< 0.0001 (vs N2)	25	68/72	32.59	
aak-1 OE	*efk-1* RNAi (L4)	19.09	0.0034	23	70/72	18.94	
N2 (WT)	EV	18.25		21	55/63		
N2 (WT)	*efk-1* RNAi (L4)	17.99	0.8715	21	51/72		
aak-1 OE	EV	24.58	< 0.0001 (vs N2)	29	67/72	34.68	
aak-1 OE	*efk-1* RNAi (L4)	23.32	0.0322	27	63/71	27.78	
N2 (WT)	EV (L4)	19.19		21	77/84		**Fig. 5f**
N2 (WT)	*efk-1* RNAi (L4)	20.38	0.523	21	66/84		
eat2	EV (L4)	24.81		29	74/84	29.29	
eat2	*efk-1-1* RNAi (L4)	23.39	0.0336	27	71/84	21.89	
N2 (WT)	EV (L4)	19.75		24	59/71		
N2 (WT)	*efk-1* RNAi (L4)	20.63	0.6443	24	75/84		
eat2	EV (L4)	24.38		30	48/70	23.44	
eat2	*efk-1-1* RNAi (L4)	18.85	0.0009	26	53/72	-4.54	
N2 (WT)	EV	20.72		24	69/84		**Fig. S4b**
N2 (WT)	*eef-2* RNAi	22.73	0.0035	26	82/94	9.65	
N2 (WT)	EV	18.59		22	62/72		**Fig. S4b**
N2 (WT)	*eef-2* RNAi	20.34	0.0213	24	62/66	9.41	
eat2	EV	23.05		31	68/90		
eat2	*eef-2* RNAi	21.83	0.275	25	89/96		
N2 (WT)	*ad libitum*	16.93		20	65/80		**Fig.5g**
N2 (WT)	*DR*	23.54	< 0.0001	28	61/80	39.04	
eef-2 OE	*ad libitum*	16.46		18	62/80		
eef-2 OE	*DR*	17.42	0.3934	22	58/80	5.83 (vs AL)	
N2 (WT)	*ad libitum*	18.57		22	69/80		
N2 (WT)	*DR*	27.4	< 0.0001	36	59/80	47.55	
eef-2 OE	*ad libitum*	15.31		18	72/84		
eef-2 OE	*DR*	20.03	0.0028	25	62/79	30.83 (vs AL)	
N2 (WT)	EV	19.65		23	78/84		**Fig. 6d**
N2 (WT)	*tiar-1* RNAi	16.96	0.0009	19	74/84		
daf-2	EV	49.25		67	43/84	150.64	
daf-2	*tiar-1* RNAi	50.57	0.6081	63	57/84	157.35	
N2 (WT)	EV	21.32		23	89/96		
N2 (WT)	*tiar-1* RNAi	18.76	0.0012	19	85/96		
daf-2	EV	53.94		67	60/96	153	
daf-2	*tiar-1* RNAi	45.54	0.0789	63	59/96	113.6	
N2 (WT)	EV	16.68		20	85/96		**Fig. 6e**
N2 (WT)	*gtbp-1* RNAi	16.69	0.8946	20	90/96		
daf-2	EV	49.41		73	66/96	196.22	
daf-2	*gtbp-1* RNAi	47.1	0.5291	65	59/96	182.37	
N2 (WT)	EV	18.04		21	93/96		
N2 (WT)	*gtbp-1* RNAi	17.66	0.6315	21	88/96		
daf-2	EV	50.52		68	71/96	180.04	
daf-2	*gtbp-1* RNAi	50.82	0.8031	66	67/96	181.71	
