# Supplementary material for: AMPK‐mediated formation of stress granules is required for dietary restriction‐induced longevity in Caenorhabditis elegans
Source: Aging Cell. 2020 May 20;19(6):e13157. doi: 10.1111/acel.13157 (PMC7294782; doi:10.1111/acel.13157)
Supplement: Supplementary file 7 — Table S2 [file ACEL-19-e13157-s007.pdf]

Extended Data Table 2 | Statistical data for *C. elegans* oxidative stress experiments

strain	treatment	mean(hours)	*p value*	75%	n
N2 (WT)	200 mM paraquat	7.5		9	72
gtbp-1(ax2029)	200 mM paraquat	5.8	< 0.0001 (vs N2)	7	72
gtbp-1(ax2055[gtbp-1::gfp])	200 mM paraquat	7.5	0.9877 (vs N2)	9	72
N2 (WT)	200 mM paraquat	8.5		10	72
gtbp-1(ax2029)	200 mM paraquat	6.8	0.0033 (vs N2)	9	72
gtbp-1(ax2055[gtbp-1::gfp])	200 mM paraquat	8.2	0.9877 (vs N2)	10	72
