# Supplementary material for: AMPK‐mediated formation of stress granules is required for dietary restriction‐induced longevity in Caenorhabditis elegans
Source: Aging Cell. 2020 May 20;19(6):e13157. doi: 10.1111/acel.13157 (PMC7294782; doi:10.1111/acel.13157)
Supplement: Supplementary file 8 — Figure Legends [file ACEL-19-e13157-s008.pdf]

Extended figures

EX Figure S1.

a. Stress granule formation was visualized by fluorescence microscopy in L1 and Day1 adult stage TIAR-1::GFP animals. Worms were treated with or without heat shock 37 ^{o}C for 15 minutes.

b. TIAR-1::GFP animals were treated with heat shock 37 ^{o}C for 15 minutes and then followed by 8 hr recovery at 20 ^{o}C. Right panels, quantitative analysis of stress granules.

c. Survival curves of wild type N2, *gtbp-1(ax2029)* ,and *gtbp-1(ax2029[gtbp-1::gfp])* mutants exposed to 200 mM paraquat in M9 buffer. Statistic for survival curves are in Supplementary table 2.

d. L3 stage *gtbp-1::gfp* animals were treated with or without heat shock 37 ^{o}C for 15 minutes.

EX Figure S2.

a. The formation of TIAR-1::GFP granules in Day 1 and Day 4 adult worms treated with *ad libitum* or 2 hour starvation.

b. The formation of GTBP-1::GFP granules in L3 stage worms treated with *ad libitum* or 2 hour starvation

c. *tiar-1::gfp* transgenic animals were fed with L4440 or *gtbp-1* RNAi bacteria from eggs. TIAR-1::GFP foci in the intestinal cells was observed under *ad libitum* conditions or 2 hr starvation.

d. Day 1 adult *tiar-1::gfp* animals were treated with *ad libitum* or 2 hr starvation and then followed by 6 hr *ad libitum* recovery at 20 ^{o}C. Right panels, quantitative analysis of stress granules for **c and d**. Each data point represents the number of stress granules per intestinal cell. Data were analyzed by one way or two way ANOVA. Levels of significance are shown as ****, $p<0.0001$.

EX Figure S3.

a. The formation of TIAR-1::GFP granules in Day 1 and Day 4 adult *eat-2;tiar-1::gfp* mutants.

b. *tiar-1::gfp* transgenic animals at L4 larval and Day 1 adult stages were fed with UV-killed *E. coli* $5X10^{10}$ cells/ml (*ad libitum*) or $1X10^{8}$ cells/ml (DR) for 16 hours.

c. Day 1 adult *tiar-1::gfp* animals were treated with *ad libitum* or 16 hr sDR and then followed by 6 hr *ad libitum* recovery at 20 ^{o}C.

EX Figure S4.

a. *tiar-1::gfp* transgenic animals grown on control or *aak-1* RNAi bacteria were treated with or without heat shock 37 ^{o}C for 15 minutes. Right panel, quantitative analysis of stress granules. Each data point represents the number of stress granules per intestinal cell. Data were analyzed by two way ANOVA. Levels of significance are shown as ****, $p<0.0001$.

b. Lifespan analysis of wild-type N2 animals (left) and *eat-2* mutants (right) grown on empty vector or *eef-2* RNAi bacteria during adulthood. Statistic for survival curves are in Supplementary table 1.

EX Figure S5.

a. The formation of GTBP-1::GFP granules in L3 stage worms fed with bacteria containing control vector or *daf-2* RNAi plasmids.

b. *eat-2* mutants carrying TIAR-1::GFP were treated with L4440 or *daf-2* RNAi from eggs. Right panels, quantitative analysis of stress granules.
